# A First-Principles Study on the Hydration Behavior of (MgO)_n_ Clusters and the Effect Mechanism of Anti-Hydration Agents

**DOI:** 10.3390/ma15103521

**Published:** 2022-05-13

**Authors:** Yu Gao, Long Dong, Liang Huang, Zhong Huang, Faliang Li, Haijun Zhang, Shaowei Zhang

**Affiliations:** 1 The State Key Laboratory of Refractories and Metallurgy, College of Materials and Metallurgy, Wuhan University of Science and Technology, Wuhan 430081, China; gaoyu@wust.edu.cn (Y.G.); dl18797318871@163.com (L.D.); huangzhong@wust.edu.cn (Z.H.); lfliang@wust.edu.cn (F.L.); 2 College of Engineering, Mathematics and Physical Sciences, University of Exeter, Exeter EX4 4QF, UK; sw-zhang2004@yahoo.co.uk

**Keywords:** MgO hydration, clusters, mechanism of hydration, anti-hydration agents, first-principles calculations

## Abstract

Magnesia-based refractory is widely used in high-temperature industries; its easy hydration is, however, a key concern in refractory processing. Understanding the hydration mechanism of MgO will help in solving its hydration problem. Herein, the hydration behavior of (MgO)_n_ (n = 1–6) at the molecular level and the effect mechanisms of several anti-hydration agents on the hydration of (MgO)_4_ were investigated with first-principles calculations. The results indicated that the following: (1) The smaller the (MgO)_n_ cluster size, the more favorable the hydration of MgO and the tendency to convert into Mg(OH)_2_ crystal; (2) Anti-hydration agents can coordinate with the unsaturated Mg atom of (MgO)_4_ to form a bond, increasing the coordination number of Mg, thus reducing its activity when reacting with H_2_O; (3) The greater the number of −COOH groups and the longer the chain length in the anti-hydration agents, the better its effect of inhibiting the hydration of MgO. These findings could enhance the understanding of the mechanism of hydration of MgO and provide theoretical guidance for the design of novel anti-hydration agents.

## 1. Introduction

Magnesia or magnesium oxide (MgO) is an important refractory raw material with the combined characteristics of high fire resistance and excellent alkaline stability. Magnesia refractory is widely used in key parts such as the converter, ladle and tundish, which are of great significance for smelting clean steel [1,2,3,4,5]. However, the hydration of magnesium oxide can cause cracks due to the density mismatch between magnesium oxide (ρ = 3.5 g/cm^3^) and its hydroxide (ρ = 2.4 g/cm^3^), which damages the integrity of the material and limits its industrial application as a refractory material [6]. At present, the mechanism of the hydration of MgO at the molecular level is still not clear, which restricts the solutions to the hydration problem of MgO [7,8,9,10,11]. Therefore, it is necessary to study the initial adsorption behavior of water molecules on magnesium oxide at the molecular scale [12,13,14,15,16].

In order to fundamentally solve the hydration problem of MgO, researchers have carried out a large number of experimental studies on hydration resistance. In the study of Chen et al., oleic acid and stearic acid were used as hydration-resistant modifiers to significantly improve the hydration resistance of magnesia refractories due to the formation of insoluble films on the surface of MgO particles [17]. Similarly, Salomão and Pandolfelli evaluated how the presence of different anti-hydration agents influences the kinetics of the MgO hydration reaction. They found that citric acid can be adsorbed on the magnesia surface, building up an insoluble magnesium citrate protective coating and inhibiting the magnesia hydration [18,19,20]. The effects of prophetic acid, acetic acid and formic acid on the hydration of magnesia were also investigated by T. dos Santos Jr. et al. [21], who reported that the hydration degree of magnesia increased according to the following sequence: propionic acid > acetic acid > formic acid > distilled water. Therefore, carboxylic acids, such as citric, malic and acetic acid, are demonstrated to influence the hydration behavior of MgO, but their mechanisms of action are not fully understood.

As an effective means by which to investigate the interface reaction, first-principles calculations can supplement the experimental explanation, so it has attracted increased attention from researchers in recent years. A cluster is considered as a bridge between molecular and crystal structures, which can be used as pre-nucleation clusters leading to the formation of minerals [22]. Clusters can also be regarded as the transitional forms between atoms and bulk, and their fundamental properties depend vitally on the cluster size [23]. Thus, the interface between magnesium oxide and water or anti-hydration agents can be extracted at the cluster level to model the interaction between them. Although the reaction interface at clusters is small, it could essentially reflect the hydration process of magnesium oxide [24].

In this study, the hydration behavior of (MgO)_n_ clusters (n = 1–6) was systematically investigated with first-principles calculations. The influences of the boric acid (BA), oxalic-acid (OA), tartaric-acid (TA), citric-acid (CA) and ISOBAM-104 (IB-104, CAS No. 52032-17-4, an amide-ammonium salt type of copolymer of isobutylene and maleic-anhydride developed by KURARAY, whose full name is poly[(isobutylene-alt-maleic acid, ammonium salt)-co-(isobutylene-alt-maleic anhydride)]) on the hydration of (MgO)_4_ clusters were evaluated, and the effect mechanism of anti-hydration agents was discussed.

## 2. Calculation Methods

All the calculations were carried out using density functional theory embedded in the DMol^3^ package (BIOVIA company, San Diego, CA, USA) [25]. Generalized gradient approximation (GGA) coupled with the Perdew-Burke-Ernzerhof (PBE) mixed exchange-correlation functional was adopted [26]. A double numerical basis set with polarization functions (DNP) was established to describe the valence electrons, and an all-electron treatment was used to perform full optimization of the investigated cluster model without symmetry constraints [27]. The convergence accuracy was set as fine quality with the tolerance for a total optimization energy of 1.0 × 10^−5^ Ha, maximum force of 2 × 10^−3^ Ha/Å, and maximum displacement of 5 × 10^−3^ Å, respectively. For the self-consistent calculation, the SCF density convergence was 1.0 × 10^−6^ e/Å^3^ and smearing was set to 5.0 × 10^−^^3^ Ha.

The lowest energy and ground-state configuration of (MgO)_n_ (n = 1–6) clusters were determined with reference to the existing literature [28,29,30,31,32,33]. The water molecules or anti-hydration agents (AA) were placed near the (MgO)_n_ (n = 1–6) clusters by gradually increasing their amounts. Next, the assumed [(MgO)_n_], [(MgO)_n_·nH_2_O], and [(MgO)_n_·mAA·(n-m)H_2_O] *(*n = 1–6, m *≤* n) structures were optimized. The COSMO solvation model (solvent is water, ε= 78.54) was used to simulate the solvent effect of free water in solution.

The average reaction energy (*E*_avg_) per MgO unit between (MgO)_n_ clusters and the water molecules is given by

*E*_avg_ = (*E*_(MgO)_n_·nH_2_O_ − (*E*_(MgO)_n__
*+ E*_H_2_O_))/n, (1)

where n is the number of water molecules in the optimized cluster, *E*_(MgO)_n__ is the total energy of the optimized (MgO)_n_ cluster, *E*_H_2_O_ is the total energy of the water molecule, and *E*_(MgO)_n_·nH_2_O_ is the total energy of the optimized (MgO)_n_·nH_2_O cluster.

Similarly, the average Gibbs free energy change (*∆G*_avg_) per MgO unit between (MgO)_n_ cluster and the water molecules is given by

*∆G*_avg_ = (*G*_(MgO)_n_·nH_2_O_ − (*G*_(MgO)_n__ + *G*_H_2_O_))/n, (2)

where *G*_(MgO)_n__ is the free energy of the optimized (MgO)_n_ clusters, *G*_H_2_O_ is the free energy of a water molecule, and *G*_(MgO)_n_·nH_2_O_ is the free energy of the optimized (MgO)_n_·nH_2_O clusters. The free energy (*G*) values are obtained with a vibrational analysis.

Negative values of *ΔG* indicate that the addition of one water molecule to the cluster is thermodynamically favorable, i.e., spontaneous.

The stepwise hydration reaction energy and Gibbs free energy change (*∆G*) are defined as

*∆E* = *E*_(MgO)_n_·mH_2_O_
*− (E*_(MgO)_n_·(m−1)H_2_O_
*+ E*_H_2_O_), *(*0 < m ≤ n*)* (3)

*∆G* = *G*_(MgO)_n_·mH_2_O_ − (*G*_(MgO)_n_·(m−1)H_2_O_ + *G*_H_2_O_), *(*0 < m ≤ n*)* (4)

The average reaction energy and Gibbs free energy change (*∆G*) for (MgO)_n_·nH_2_O clusters conversion to Mg(OH)_2_ Crystal were defined as

*∆E* = [*E*_nMg(OH)_2__ − *E*_(MgO)_n_·nH_2_O_]/n, (5)

*∆G* = [*G*_nMg(OH)_2__ − *G*_(MgO)_n_·nH_2_O_]/n, (6)

The energy change caused by the addition of one anti-hydration agent or water molecule to the cluster is defined as

*∆E* = *E*_(MgO)_n_·xAA*·*_− *(E*_(MgO)_n_·(x−1)AA*·*_*+ E*_AA_*),* (0 < x ≤ n) (7)

*∆E* = *E*_(MgO)_n_·xAA·yH_2_O_ − *(E*_(MgO)_n_·xAA·(y−1)H_2_O_
*+ E*_H_2_O_*),* (0 < x + y ≤ n) (8)

## 3. Results

### 3.1. Hydration Mechanism of (MgO)_n_·nH_2_O Clusters

**Figure 1:**
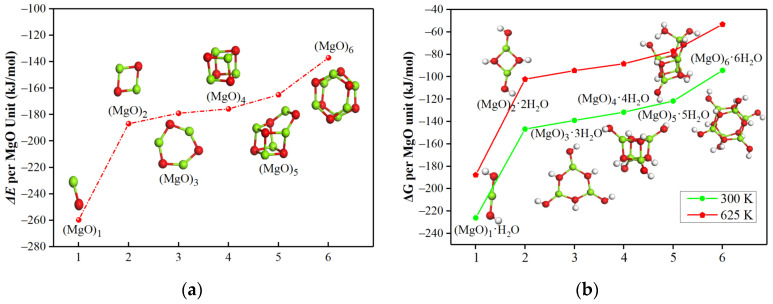
The lowest energy structures of (MgO)_n_ (n = 1–6) and (MgO)_n_·nH_2_O clusters, (**a**) the reaction energy, and (**b**) Gibbs free energy change for the reaction between (MgO)_n_ clusters and n H_2_O molecules. [Legend: red, O atoms; Green, Mg atoms; white, and H atoms].

The optimized geometries of (MgO)_n_·(n = 1–6) and (MgO)_n_·nH_2_O clusters are shown in Figure 1, which are consistent with previous reports [28,29,30,31,32,33]. In the hydration reaction process, one (MgO)_n_ can react with H_2_O to form the corresponding hydration products-(MgO)_n_·nH_2_O. The average reaction energy (*E*_avg_) per MgO unit between (MgO)_n_ clusters and n H_2_O molecules is plotted in Figure 1a. It was found that the reaction of (MgO)_n_ clusters with H_2_O is exothermic and the released heat per MgO unit decreased with an increase in cluster size. The average Gibbs free energy change (*∆G*_avg_) per MgO unit of the reaction between (MgO)_n_ clusters and H_2_O molecules (as shown in Figure 1b) was negative at room temperature (300 K) and at an elevated temperature (625 K), further demonstrating that (MgO)_n_ clusters were able to easily react with H_2_O. The smaller the (MgO)_n_ cluster size, the more favorable the reaction between (MgO)_n_ clusters and H_2_O. The possible reason is that the smaller the (MgO)_n_ cluster size, the smaller the coordination number of the Mg atom, resulting in increased activity to dissociate the water.

**Figure 2:**
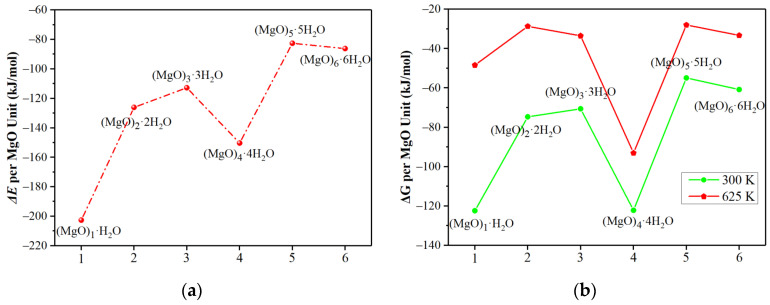
(**a**) The reaction energy and (**b**) Gibbs free energy change for conversion of (MgO)_n_·nH_2_O clusters into Mg(OH)_2_ crystals.

The tendency for the conversion of hydration products − (MgO)_n_·nH_2_O to Mg(OH)_2_ crystal was also investigated, as shown in Figure 2. The results showed that the conversion of (MgO)_n_·nH_2_O into Mg(OH)_2_ crystal was also an exothermic reaction. Generally, the released heat per MgO unit decreased with an increase in the (MgO)_n_·nH_2_O cluster size when it was converted into a Mg(OH)_2_ crystal, except for (MgO)_4_·4H_2_O. The Gibbs free energy change per MgO unit for the conversion of (MgO)_n_·nH_2_O to Mg(OH)_2_ crystal at different cluster sizes were negative at room temperature (300 K) and at an elevated temperature (625 K). It indicated that the hydrated (MgO)_n_·nH_2_O clusters tend to be transformed into Mg(OH)_2_ crystals spontaneously. Among them, (MgO)_4_·4H_2_O clusters show a tendency to form Mg(OH)_2_ crystals more easily from the point of view of reaction energy and Gibbs free energy change. One possible reason is that the (MgO)_4_ cluster helps to maintain the cube structure, which is closer to the MgO crystal structure than other clusters in terms of crystal structure. Therefore, (MgO)_4_ cluster can better reflect the hydration behavior of MgO. The above results indicated that the smaller the (MgO)_n_ cluster size, the more favorable the hydration of MgO and the trend to convert into Mg(OH)_2_ crystal, which is consistent with the fact that the rate of the hydration reaction increases as the crystallinity of the MgO decreases (i.e., smaller mean crystallite size) [34].

**Figure 3:**
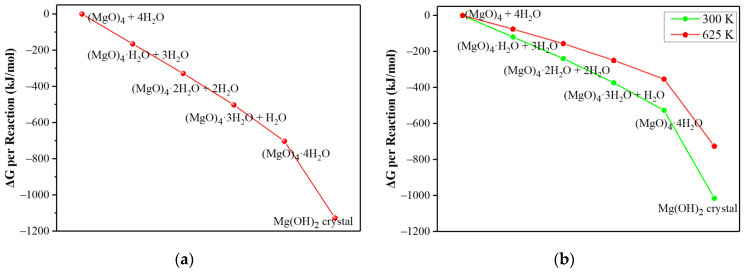
(**a**) The reaction heat (**b**) and reaction free energy for the stepwise hydration of (MgO)_4_.

Taking (MgO)_4_ clusters as an example, its stepwise reaction with the H_2_O molecule was also studied in detail to expound the hydration characteristics of MgO, as shown in Figure 3. As the reaction progressed, a great deal of heat was released at each step, and the reaction energies were −165.0, −163.2, −173.9 and −200.6 kJ/mol, respectively. The Gibbs free energy changes of each step at 300 K were −119.1, −120.4, −134.3 and −152.9 kJ/mol, respectively. Although the Gibbs free energy change at 625 K moved towards a positive direction, it was still less than zero. The reaction energy for the conversion of (MgO)_4_·4H_2_O to Mg(OH)_2_ was −425.1 kJ/mol, and Gibbs free energy changes were −488.9 kJ/mol at 300 K and −372.6 kJ/mol at 625 K, respectively. These results show that (MgO)_4_ clusters were easily and gradually hydrated and transformed into Mg(OH)_2_ crystals at room temperature (300 K) and at an elevated temperature (625 K).

### 3.2. Effect Mechanism of Anti-Hydration Agents on Hydration of (MgO)_4_ Cluster

The previous study suggested that the hydration of magnesium oxide could be inhibited by the addition of various additives to a certain degree [17,18,19,20,21,35]. To investigate the potential of boric acid (BA), oxalic acid (OA), tartaric acid (TA), citric acid (CA) and ISOBAM-104 (IB-104) as anti-hydration agents, the effect of these molecules on the hydration of (MgO)_4_ cluster was investigated.

Without the addition of an anti-hydration agent, the first H_2_O was dissociated spontaneously on the (MgO)_4_ clusters to form Mg–OH and an O–H bond, because the unsaturated Mg ion of (MgO)_4_ had an empty orbital, and the oxygen atom of H_2_O contained lone pairs of electrons for donation. The reaction between the first H_2_O and (MgO)_4_ cluster to form (MgO)_4_·H_2_O occurred as an exothermic reaction that released −139.96 kJ/mol of energy.

#### 3.2.1. Effect Mechanism of Boric Acid on Hydration of (MgO)_4_ Cluster 

**Figure 4:**
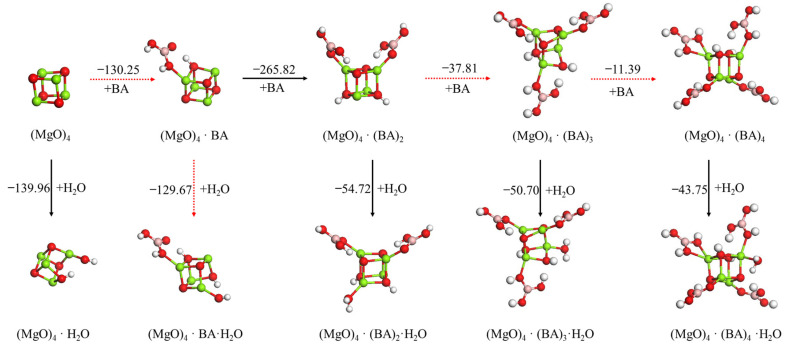
Optimized structures and relative energies of (MgO)_4_·(BA)_n_ (n = 0–4) and (MgO)_4_·(BA)_n_·H_2_O (n = 0−4). [Legend: red, O atoms; green, Mg atoms; pink, B atoms; white, H atoms; gray, C atoms.]. The black arrow represents the preferred reaction path.

The effect of the addition of boric acid content on the hydration reaction of the (MgO)_4_ cluster was investigated, as shown in Figure 4. When first boric acid molecule was adsorbed on the (MgO)_4_ cluster, the –OH of boric acid dissociated to form a Mg–OH and O–H bond on the (MgO)_4_ cluster. The dissociation adsorption energy was −130.25 kJ/mol, which is slightly more positive than adsorption energy of the first H_2_O on the (MgO)_4_ cluster, indicating that a small amount of boric acid cannot inhibit the initial hydration of (MgO)_4_. After the (MgO)_4_ cluster adsorbed one boric acid, the second H_2_O was adsorbed and dissociated on (MgO)_4_·BA, and the dissociation adsorption energy changed from −139.96 kJ/mol to −129.67 kJ/mol. When the second boric acid was adsorbed on the (MgO)_4_·BA cluster, the –OH of boric acid also dissociated to form a Mg–OH and O–H bond with a dissociation adsorption energy of –265.82 kJ/mol, indicating that the (MgO)_4_·BA cluster preferentially adsorbs the second boric acid instead of the second H_2_O. When the (MgO)_4_ cluster adsorbed two or more boric acid molecules, the H_2_O was only adsorbed in its molecular form and no longer dissociated on (MgO)_4_·(BA)_n_ (n = 2–4). Nevertheless, (MgO)_4_·(BA)_n_ preferentially adsorbed another H_2_O instead of a boric acid molecule after (MgO)_4_ adsorbed two or more boric acid molecules, because the absolute value of boric acid’s adsorption energies was smaller than that of H_2_O’s. Therefore, increasing the amount of boric acid can retard the hydration of (MgO)_4_ but cannot completely inhibit its hydration.

#### 3.2.2. Effect Mechanism of Oxalic Acid on Hydration of (MgO)_4_ Cluster

**Figure 5:**
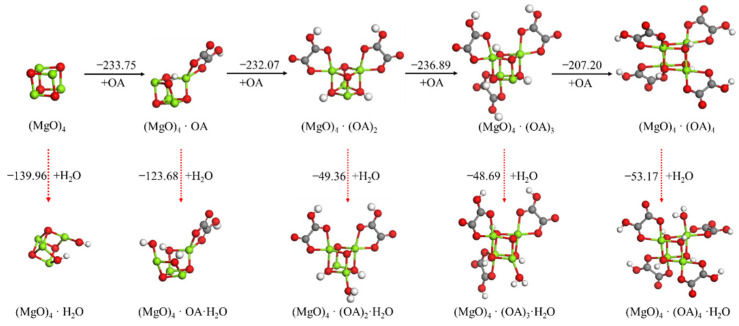
Optimized structures and relative energies of (MgO)_4_·(OA)_n_ (n = 0−4) and (MgO)_4_·(OA)_n_·H_2_O (n = 0−4). [Legend: red, O atoms; Green, Mg atoms; white, and H atoms; Gray, C atoms.]. The black arrow represents the preferred reaction path.

The effect of the addition of oxalic acid on the hydration reaction of the (MgO)_4_ cluster was investigated, as shown in Figure 5. When the first oxalic acid molecule was adsorbed on the (MgO)_4_ cluster, the –OH of oxalic acid also dissociated to form Mg–OH and an O–H bond on the (MgO)_4_ cluster. However, its dissociation adsorption energy was –233.75 kJ/mol, which is much smaller than the adsorption energy of the first H_2_O on the (MgO)_4_ cluster, indicating that the oxalic acid was preferentially adsorbed on the (MgO)_4_ clusters and inhibited the initial hydration of (MgO)_4_. Similarly, the (MgO)_4_·OA clusters will preferentially adsorb the second oxalic acid instead of the second H_2_O. When two or more oxalic acid molecules were adsorbed on the (MgO)_4_ clusters, H_2_O was no longer dissociated on (MgO)_4_·(OA)_n_ (n = 2–4), but was instead adsorbed in the form of molecules. Meanwhile, the absolute value of oxalic acid’s adsorption energies was larger than that of H_2_O’s, suggesting that (MgO)_4_·(OA)_n_ preferentially adsorbs another oxalic acid instead of the H_2_O molecule after (MgO)_4_ adsorbing two or more oxalic acid molecules. It showed that an appropriate amount of oxalic acid can protect (MgO)_4_ clusters and inhibit their hydration. With an increase in the adsorption amount of oxalic acid, the hydration-inhibition effect is improved.

#### 3.2.3. Effect Mechanism of Tartaric Acid, Citric Acid and ISOBAM-104 on Hydration of (MgO)_4_ Cluster 

**Figure 6:**
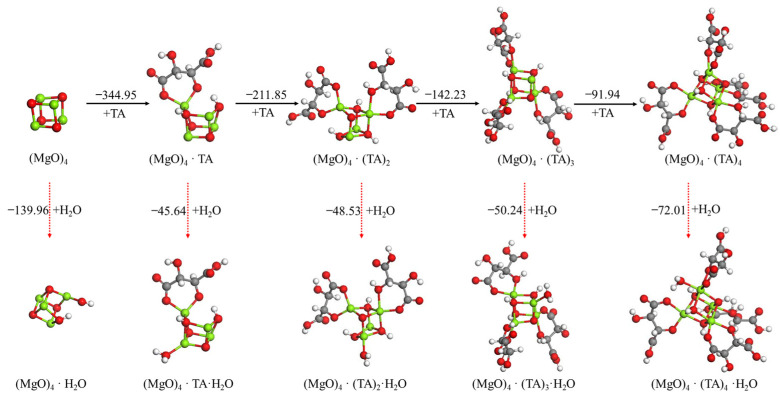
Optimized structures and relative energies of (MgO)_4_·(TA)_n_ (n = 0−4) and (MgO)_4_·(TA)_n_·H_2_O (n = 0−4). [Legend: red, O atoms; green, Mg atoms; white, H atoms; gray, C atoms.]. The black arrow represents the preferred reaction path.

**Figure 7:**
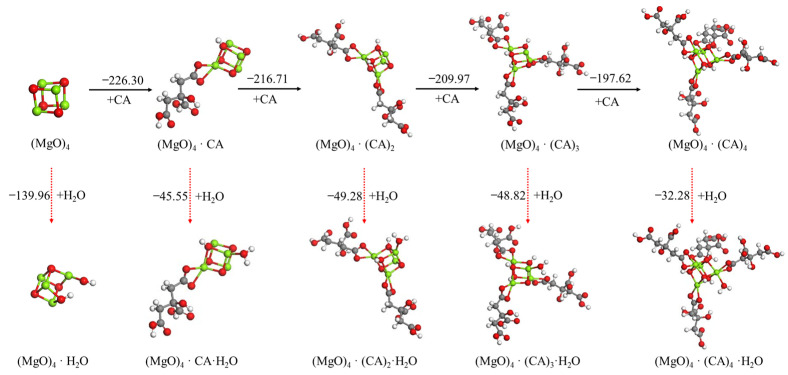
Optimized structures and relative energies of (MgO)_4_·(CA)_n_ (n = 0−4) and (MgO)_4_·(CA)_n_·H_2_O (n = 0−4). [Legend: red, O atoms; green, Mg atoms; white, H atoms; gray, C atoms.]. The black arrow represents the preferred reaction path.

**Figure 8:**
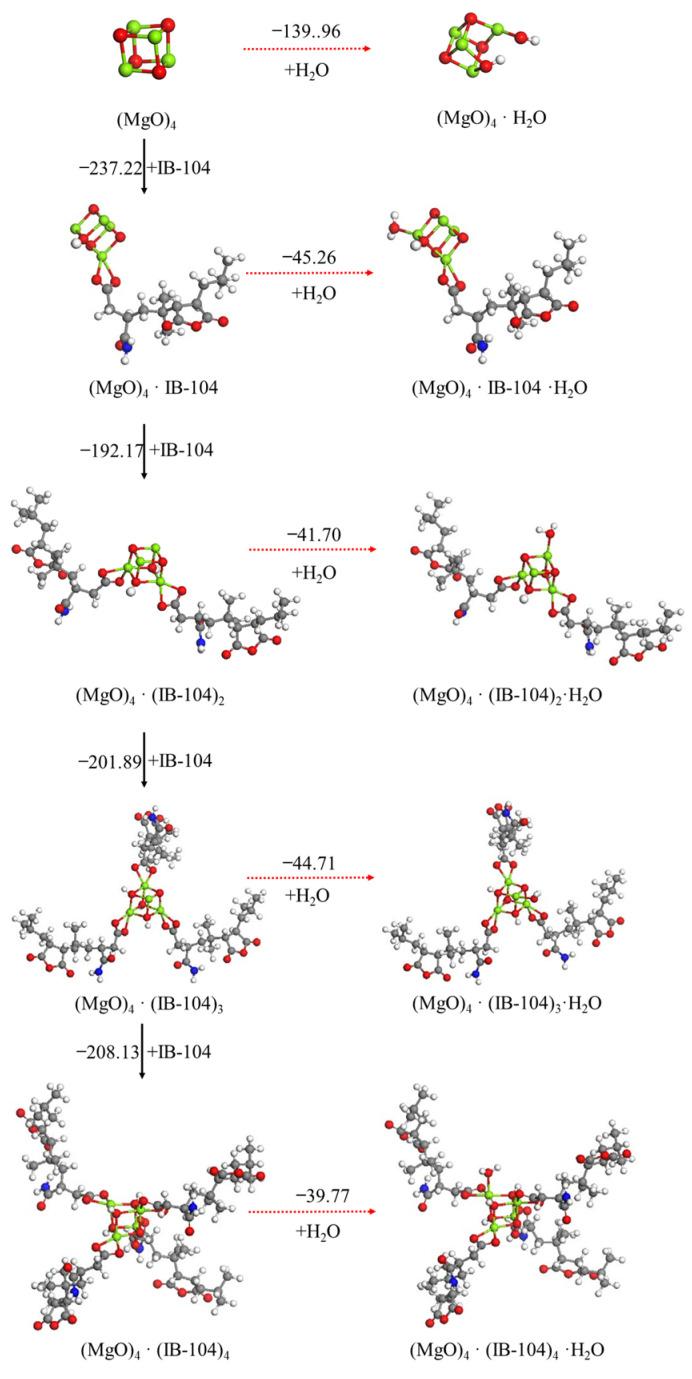
Optimized structures and relative energies of (MgO)_4_·(IB-104)_n_ (n = 0−4) and (MgO)_4_·(IB-104)_n_·H_2_O (n = 0−4). [Legend: red, O atoms; green, Mg atoms; white, H atoms; gray, C atoms; blue, N atoms.]. The black arrow represents the preferred reaction path.

The effect of the use of tartaric acid, citric acid or ISOBAM-104 as the anti-hydration agent (AA) and its content on the hydration reaction of the (MgO)_4_ cluster was also investigated, as shown in Figure 6, Figure 7, Figure 8. A similar phenomenon to oxalic acid was observed. The first tartaric acid, citric acid or ISOBAM-104 preferentially adsorbed on the (MgO)_4_ clusters and inhibited the initial hydration of (MgO)_4_. Nevertheless, when only one tartaric acid, citric acid or ISOBAM-104 molecule was adsorbed on the (MgO)_4_ clusters, H_2_O was no longer dissociated on (MgO)_4_·(AA)_n_ (n = 2–4), but adsorbed in the form of a molecule, indicating that acid, citric acid or ISOBAM-104 has a better ability to inhibit the initial hydration of (MgO)_4_. Meanwhile, the adsorption energies also suggested that (MgO)_4_·(AA)_n_ preferentially adsorbs another tartaric acid, citric acid or ISOBAM-104 instead of an H_2_O molecule after (MgO)_4_ by adsorbing one or more anti-hydration agent molecules. It is worth mentioning that the affinity of citric acid or ISOBAM-104 to MgO was stronger than that of H_2_O molecule. The results showed that a small amount of tartaric acid, citric acid or ISOBAM-104 can protect (MgO)_4_ clusters and inhibit their hydration. With the increase in the adsorption amount of tartaric acid, citric acid or ISOBAM-104, the hydration inhibition effect obviously improved.

## 4. Discussion

The thermodynamic data of the reaction between (MgO)_n_ clusters and H_2_O molecules demonstrated that the smaller the size of MgO, the more easily it is hydrated. The hydrated (MgO)_n_·nH_2_O clusters show a tendency to convert to Mg(OH)_2_ crystals, among which (MgO)_4_·4H_2_O is easier to convert to Mg(OH)_2_ crystals from the point of view of reaction energy and reaction free energy. Each hydration step of the (MgO)_4_ clusters releases a lot of heat, and the reaction free energy is negative at 300 K and 625 K, indicating that (MgO)_4_ clusters are easily hydrated step by step, and the hydrated (MgO)_4_·4H_2_O tends to transform into Mg(OH)_2_ crystals.

Different kinds of anti-hydration agents and H_2_O are competitively adsorbed on (MgO)_4_ clusters. Even the amount of adsorbed boric acid molecules increased to more than two and the (MgO)_4_·(BA)_n_ preferentially adsorbed another H_2_O instead of a boric acid molecule, suggesting that boric acid could not completely inhibit the initial hydration of (MgO)_4_ clusters. By increasing the adsorption amount of oxalic acid to two or more, the H_2_O was adsorbed on the (MgO)_4_ cluster in the form of a molecule, and the adsorption of another oxalic acid molecule was more favorable than the adsorption of the H_2_O molecule. After only one molecule of tartaric acid, citric acid or ISOBAM-104 was adsorbed on the (MgO)_4_ cluster, H_2_O was adsorbed in the form of a molecule instead of the occurrence of dissociation. With the increase in the adsorption amount, the adsorption energy of tartaric acid, citric acid or ISOBAM-104 was found to be more negative than that of H_2_O, indicating the preferential adsorption of tartaric acid, citric acid or ISOBAM-104. These results indicated the following: (1) the inhibition effect of boric acid on (MgO)_4_ hydration was not obvious because borate is a weak ligand; (2) the adequate amount of oxalic acid could protect the (MgO)_4_ cluster and inhibit its hydration to some extent; (3) the tartaric acid, citric acid or ISOBAM-104 had a good inhibition effect on (MgO)_4_ hydration. By increasing the dosage of tartaric acid, citric acid or ISOBAM-104, the inhibition effect would improve. At pH~5, the hydration rate was proportional to the proton concentration [35]. On one hand, the dissociation of a water molecule is related to the deprotonation of bound water, meaning that the deprotonation of water at an acidic pH is unfavorable, whereas it is favorable at a neutral (water) or slightly basic pH (boric acid). On the other hand, the formation of an O-H bond (formed by releasing of H+ species) is favored when the acidity coefficient (pKa) of organic acid is stronger [36]. At a pH < 5, the rate-controlling step was proton attack [MgO + 2H^+^ → Mg^2+^ + H_2_O] and depended on the concentration of Mg^2+^ [35]. In this situation, the strong bonding energy (adsorption energy) between the ligand of the anti-hydration agent can chelate Mg^2+^ and suppress the formation of Mg(OH)_2_. Simply, suitable organic acids could have a high chemical affinity to bind to Mg ions and protect them from hydration. Once a certain supersaturation threshold is reached, the “unwanted” Mg(OH)_2_ formation can be avoided.

By analyzing the molecular structure of the anti-hydration agents, we found the following: (1) the greater the number of −COOH groups in the anti-hydration agent, the more easily it bonds with the unsaturated Mg atom of the (MgO)_4_ cluster; (2) the greater the molecular weight of the anti-hydration agent, the better its protective effect is on the (MgO)_4_ cluster; thus, it can better inhibit the hydration of MgO. When tartaric acid, citric acid or ISOBAM-104 is used as the anti-hydration agent, it can hinder the spontaneous dissociation of H_2_O on (MgO)_4_ and even if initially only one anti-hydration agent molecule is adsorbed, it can inhibit the hydration of MgO.

The calculation results are consistent with Li and Pandolfelli’s experimental results demonstrating that tartaric acid and citric acid could inhibit the hydration of MgO to a certain extent, although citric acid is more effective than tartaric acid [18,37]. However, as of yet, there is no report describing the effect of ISOBAM-104 as an anti-hydration agent. Therefore, the use of ISOBAM-104 to modify MgO-containing materials in the future is expected to improve their hydration resistance.

## 5. Conclusions

In this work, the hydration behavior of (MgO)_n_ clusters (n = 1–6) and the effect mechanism of anti-hydration agents on the hydration of (MgO)_4_ clusters were studied with first-principles calculations. The thermodynamic data of the reaction between (MgO)_n_ clusters and H_2_O molecules demonstrate that the smaller the size of MgO, the more easily it is hydrated. Each hydration step of (MgO)_4_ clusters releases a considerable amount of heat, and the reaction free energy is negative at 300 K and 625 K, indicating that the (MgO)_4_ cluster can easily be hydrated step by step. The hydrated (MgO)_n_·nH_2_O clusters show a tendency to convert to Mg(OH)_2_ crystals, among which (MgO)_4_·4H_2_O is easier to convert to Mg(OH)_2_ crystals from the point of view of reaction heat and reaction free energy.

Different kinds of anti-hydration agents and H_2_O are competitively adsorbed on (MgO)_4_ clusters. The adsorption energies show that tartaric acid, citric acid or poly[(isobutylene-alt-maleic acid, ammonium salt)-co-(isobutylene-alt-maleic anhydride)] (ISOBAM-104) could protect the (MgO)_4_ cluster and has a better inhibitory effect on the hydration of (MgO)_4_ clusters, and the inhibition effect improves with increased amounts of anti-hydration agents. The greater the number of −COOH groups in the anti-hydration agents, the more easily it bonds with unsaturated Mg^2+^ on the (MgO)_4_ clusters; and the greater the molecular weight of the anti-hydration agents, the better the protective effect on the (MgO)_4_ cluster; thus it can better inhibit the hydration of MgO.

This study not only reveals the hydration mechanism behavior of MgO at the molecular level and the effect mechanism of anti-hydration agents on its hydration, but it also serves as indispensable knowledge for future experimental approaches.

## Data Availability

All data generated in this study are available from the corresponding author (L.H., huangliang1986@wust.edu.cn) without restriction.
